# Untapped potential: exploring clinical pharmacists as antibiotic stewardship ambassadors

**DOI:** 10.1017/ash.2025.69

**Published:** 2025-04-28

**Authors:** Esther Esadah, Hayden T. Schwenk, Laura L. Bio

**Affiliations:** 1 Department of Pharmacy, Children’s National Hospital, Washington, DC, USA; 2 Department of Pediatrics, Division of Infectious Diseases, Stanford School of Medicine, Stanford, CA, USA; 3 Department of Pharmacy, Lucile Packard Children’s Hospital Stanford, Palo Alto, CA, USA

## Abstract

**Objective:**

To describe inpatient clinical pharmacists’ interventions on injectable antibiotics and assess their impact on prospective audit and feedback (PAF) by the antimicrobial stewardship program (ASP).

**Design:**

Retrospective cohort study.

**Setting:**

Freestanding, quaternary-care, pediatric and obstetric hospital.

**Methods:**

We identified all clinical pharmacist interventions (iVents) documented on injectable antibiotics from November 1, 2020, through October 31, 2022. PAF performed on injectable antibiotics during the same timeframe was captured. We reported characteristics of clinical pharmacist iVents on injectable antibiotics. We also compared the incidence of PAF recommendations (PAFR) between PAF cases with prior iVent documentation for the same patient and antibiotic and those without preceding iVent documentation.

**Results:**

A total of 5,277 iVents were documented on injectable antibiotic orders. Cefazolin had the highest volume of iVents (13%). Antibiotic dose optimization was the most frequent iVent type (34%). A total of 5,152 PAF were documented by ASP pharmacists on injectable antibiotics during the study period, with 1,782 (34%) resulting in a PAFR. A total of 999 PAF (19%) had a prior iVent; 4,153 PAF did not. Comparing the two groups, the incidence of a PAFR was significantly higher in the PAF with prior iVent group compared to the PAF without prior iVent group (383/999, 38% vs 1,399/ 4,153, 34%; *P* = 0.006). Antibiotic discontinuation was the most common type of PAFR in both groups.

**Conclusions:**

Clinical pharmacists serve as ASP ambassadors, intervening on injectable antibiotic orders to improve prescribing. Future efforts to expand and incorporate clinical pharmacists in ASP initiatives are warranted.

## Introduction

Pharmacists’ expertise is recognized as an essential element of antimicrobial stewardship programs (ASPs).^1–3^ While much of the attention has focused on infectious diseases (ID)-trained pharmacists, all clinical pharmacists, regardless of their clinical setting or title, are capable of contributing to stewardship initiatives.^4,5^

Prospective audit and feedback (PAF) – the retrospective evaluation of prescribed antibiotics – is recognized as a core ASP strategy by the Infectious Diseases Society of America and the Society for Healthcare Epidemiology of America.^6^ While the method and procedures for performing PAF may vary by institution and range from formal handshake rounds to tele-stewardship, the overarching goal remains the optimization of antibiotic prescriptions.^7,8^ Additional benefits include reductions in antibiotic resistance and the incidence of *Clostridioides difficile* infection.

There are limited data on the types of interventions identified by clinical pharmacists at hospitals with an ASP. The primary objective of this study was to characterize the types of interventions (iVents) made by clinical pharmacists on injectable antibiotic orders in a pediatric hospital setting. Additionally, we sought to determine whether the incidence of ASP PAF recommendations (PAFR) differed based on the presence of a preceding clinical pharmacist iVent.

## Methods

### Study setting

Lucile Packard Children’s Hospital Stanford is a university-affiliated, 413-bed hospital that delivers quaternary-level pediatric and obstetric care. Our pharmacy practice model consists of clinical pharmacists responsible for order verification, medication preparation, or participation in family-centered rounds, based on their training and expertise. All inpatient antibiotic orders are evaluated by a clinical pharmacist for appropriateness during order review and verification. During this review, pharmacists may identify interventions to improve antibiotic prescribing. Interventions identified during order verification or rounding are documented as iVents attached to the medication order in the electronic health record (EHR) (Epic Systems Corporation Verona, Wisconsin). Content documented within an iVent includes the intervention type and subtype (Supplemental Table 1).

**Table 1.** Distribution of iVents by unit type

Clinical pharmacist recoded interventions, n (%)	Total n = 5,277	Acute Care n = 3,252	Critical Care n = 2,025
Optimize dose regimen	1811 (34.3)	1005 (30.9)	806 (39.8)
Clarify indication/plan	801 (15.2)	538 (16.5)	263 (13)
Drug administration	756 (14.3)	521 (16)	235 (11.6)
Discontinue the antibiotic	696 (13.2)	430 (13.2)	266 (13.1)
Monitoring	311 (5.7)	168 (5.2)	143 (11.7)
Medication reconciliation	209 (4)	103 (3.2)	106 (5.2)
Intravenous to enteral conversion	151 (2.9)	114 (3.5)	37 (1.8)
Change antibiotic	123 (2.3)	73 (2.2)	50 (2.5)
Allergy and adverse drug evaluation	95 (1.8)	97 (3.0)	25 (1.2)
Drug-drug interaction	23 (0.4)	14 (0.4)	9 (0.4)
Other(s)*	274 (5.2)	189 (5.8)	85 (4.2)

* Refer to Supplemental Table 1 for breakdown of iVent recommendation type of other.

Our ASP team includes two physicians trained in pediatric ID and two full-time clinical pharmacy specialists who are board-certified in ID by the American College of Clinical Pharmacy.^9^ Our ASP pharmacists perform PAF every weekday, which includes a review of all inpatient orders for injectable antibiotics that have been active for at least 48 hours.^10^ All PAFs performed, regardless of whether a recommendation is identified or not, are documented in the EHR through a customized smart form. Recommendations identified during PAF are communicated to the primary team through their rounding clinical pharmacist or directly to the team if no clinical pharmacist is present. This strategy allows for a collaborative practice and bidirectional education between the clinical and ASP pharmacists.

### Study design

This was a single-center retrospective review of interventions documented as iVents by clinical pharmacists on inpatient orders for injectable antibiotics from November 1, 2020, through October 31, 2022. All iVents documented on an injectable antibiotic order were included, as long as the iVent type was unique. PAF and PAFR on injectable antibiotics during this timeframe were also captured. iVents and PAF were excluded if data was missing from the documentation record. Given that obstetric services are excluded from the PAF program, iVents documented on obstetric antibiotic orders were excluded. Data captured from the iVent report included patient identifiers, antibiotic name, intervention date and time, intervention type and subtype, and, if available, intervention outcome. PAF data included patient identifiers, antibiotic name, audit date and time, and, if applicable, recommendation type and recommendation acceptance. This project was reviewed by Stanford University’s institutional review board and determined to be local quality improvement work that did not meet the definition of human subjects’ research.

### Study analysis

Descriptive statistics were used to characterize iVents. We matched PAF and iVents based on patient identifiers, antibiotic name, and time between the PAF and iVent. A PAF was classified as linked to an iVent (ie, PAF with prior iVent group) if they were documented for the same patient and the same antibiotic and the PAF was documented within 4 days following the iVent. A 4-day window was selected to capture iVents documented during weekends or holidays with subsequent PAF on the next business day. Due to the difference in documentation terminology for iVents and PAFR, interventions and recommendations were translated to a recoded recommendation type based on similar intent to enable comparisons (Supplemental Tables 1 and 2). Comparison of the proportion of PAFR between the PAF with prior iVent group and PAF without prior iVent group was performed using the χ^2^ test in SPSS (2021 IBM SPSS Statistics for Windows, Version 28.0. Armonk, New York: IBM Corp) with alpha set at 0.05.

**Table 2.** Comparison of iVents and antimicrobial stewardship program pharmacist PAFR, by antibiotic

Injectable antibiotic (%)	iVents n = 5,277	PAFR n = 1,782
Cefazolin	716 (13.6)	114 (6.4)
Cefepime	708 (13.4)	226 (12.7)
Ceftriaxone	594 (11.3)	155 (8.7)
Piperacillin-tazobactam	548 (10.4)	170 (9.5)
Meropenem	461 (8.7)	224 (12.6)
Vancomycin	407 (7.7)	244 (13.7)
Ampicillin	341 (6.5)	87 (4.9)
Gentamicin	237 (4.5)	58 (3.4)
Metronidazole	227 (4.3)	67 (3.8)
Ampicillin-sulbactam	209 (4)	71 (4)
Ceftazidime	128 (2.4)	64 (3.6)
Nafcillin	101 (1.9)	27 (1.5)
Ciprofloxacin	82 (1.6)	22 (1.2)
Clindamycin	75 (1.4)	40 (2.2)
Sulfamethoxazole-trimethoprim	72 (1.4)	32 (1.8)
Levofloxacin	63 (1.2)	28 (1.6)
Azithromycin	62 (1.2)	46 (2.6)
Cefoxitin	41 (0.8)	9 (0.5)
Penicillin	32 (0.6)	16 (0.9)
Tobramycin	29 (0.5)	6 (0.3)
Linezolid	24 (0.5)	0 (0)
Other*	120 (2.3)	76 (4.3)

* Other antibiotics included for iVents: ceftaroline fosamil (n=18), daptomycin (n=18), rifampin (n = 18), erythromycin (n = 16), ertapenem (n = 15), amikacin (n = 7), ceftolozane-tazobactam (n = 8), doxycycline (n=7), ceftazidime-avibactam (n = 6), imipenem-cilastatin (n = 2), tedizolid (n = 2), aztreonam (n = 1), cefuroxime (n = 1), moxifloxacin (n = 1). Other antibiotics included for PAFR: ceftaroline fosamil (n=11), daptomycin (n = 11), rifampin (n = 11), erythromycin (n = 9), ertapenem (n = 1), amikacin (n = 7), ceftolozane-tazobactam (n = 3), doxycycline (n = 4), imipenem-cilastatin (n = 3).

PAFR, Prospective audit and feedback recommendations.

## Results

**Figure 1.**
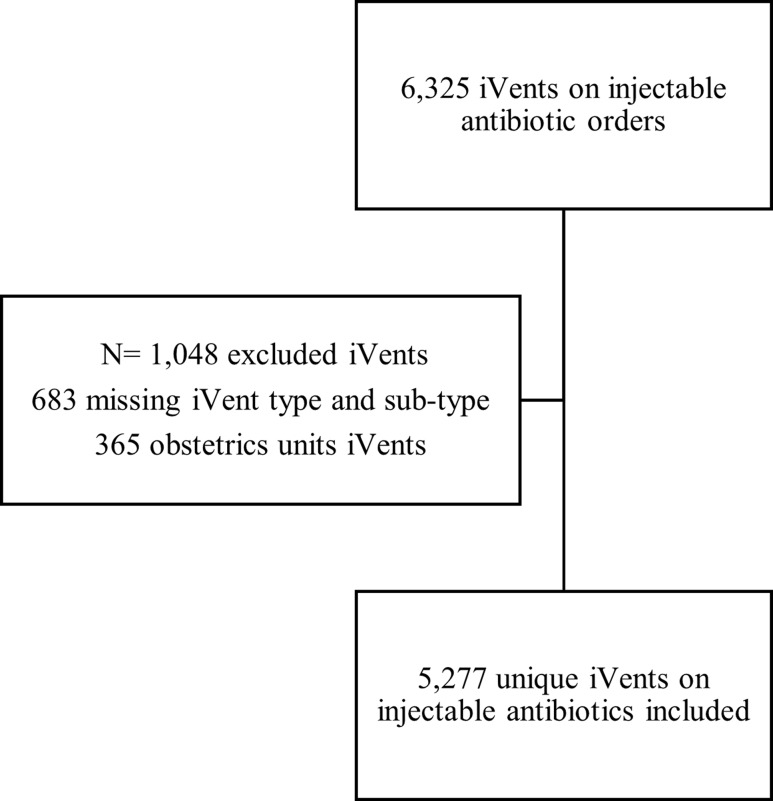
Flowchart of iVent screening.

During the study period, there were 29,753 inpatient orders for an injectable antibiotic, excluding orders from the obstetric units. A total of 6,325 iVents were documented on antibiotic orders. Application of our inclusion/exclusion criteria resulted in a cohort of 5,277 unique iVents on injectable antibiotics for analysis (Figure 1). Less than half of iVents included outcome documentation (2,324/5,277, 44%) with 1,731 (74%) iVents documented as accepted, 29 (1%) as rejected, and 564 (24%) documented as informational or non-applicable. The majority of iVents were documented on antibiotic orders from an acute care unit (Table 1). Cefazolin had the highest volume of iVents (716, 13%). The most frequent type of iVent was antibiotic dose optimization (1,811, 34%; Table 2).

A total of 5,152 PAF were documented by ASP pharmacists on injectable antibiotics during the study period, resulting in 1,782 (35%) PAFR. Vancomycin had the highest volume of PAFR (244/1,782, 14%) and the most common PAFR type was to discontinue the antibiotic (445/1,782, 25%; Table 2 and Supplemental Table 2). A total of 999 PAF (19%) had a preceding iVent documented for the same patient and antibiotic; the remaining 4,153 PAF did not have a prior iVent identified. Comparing the two groups, the PAF with prior iVent group had a significantly higher proportion of PAFR than the PAF without prior iVent group (383/999, 38% vs 1,399/ 4,153, 34%, *P* = 0.006; Table 3). Antibiotic discontinuation was the most common type of PAFR, regardless of prior iVent, accounting for 24% (91/383) of recommendations in the PAF with prior iVent group and 25% (354/1,399) in the PAF without prior iVent group (Table 3). Dose optimization was also similar between groups (16% [63/383] of PAF had a PAFR in the PAF with prior iVent group vs 14% [192/1,399] in the PAF without prior iVent group).

**Table 3.** Type of prospective audit and feedback recommendation in the presence and absence of prior iVents

Recommendation type, n (%)	PAFR with iVent n = 383	PAFR without iVent n = 1,399
Change antibiotic	58 (15.1)	211 (15.8)
Clarify	18 (4.7)	73 (5.2)
Consult recommended	26 (6.8)	119 (8.5)
Discontinue the antibiotic	91 (23.8)	354 (23.3)
Duration modification	75 (19.6)	238 (17.0)
Intravenous to enteral conversion	35 (9.1)	156 (11.2)
Monitoring	14 (3.7)	45 (3.2)
Optimize dose regimen	63 (16.4)	192 (13.7)
Other(s)*	3 (0.8)	11 (0.8)

* Refer to supplemental table 1 and 2 for breakdown of PAFR type of other.

PAFR, Prospective audit and feedback recommendations.

No statistically significant difference was detected between groups based on recommendation type per χ² test with alpha set at 0.05.

## Discussion

We report the first pediatric study describing the types of antibiotic stewardship interventions identified by clinical pharmacists and their impact on subsequent ASP PAFR. Our study demonstrated that clinical pharmacists perform many antibiotic interventions as ASP ambassadors, with most interventions focused on antibiotic dose optimization. Such interventions may improve patient safety by ensuring that patients are receiving the appropriate antibiotic dose based on their indication, their renal function, and their clinical status. Our findings align with previous studies that identified dose adjustment as one of the most frequent pharmacist interventions on antibiotic orders for adult patients, accounting for approximately two-thirds of interventions.^11^

We discovered that the types of interventions by clinical pharmacists differed from ASP pharmacist PAFR. The most common clinical pharmacist interventions were dose optimization, order clarification, and drug administration. Our ASP pharmacists’ most common recommendations were antibiotic discontinuation, therapy modification, and duration of therapy suggestions. There may be multiple reasons why the types of interventions differ between clinical and ASP pharmacists. A critical determinant of clinical intervention is the available clinical data, including microbiologic information. The difference in available information at the time of order verification vs ASP review may impact the scope and depth of pharmacist interventions. For example, at the time of antibiotic order entry, a clinical pharmacist could recommend dose optimization, and subsequently an ASP pharmacist performing PAF 48–72 hours later could recommend discontinuing the therapy based on negative culture data.

The difference in intervention types made by clinical and ASP pharmacists may also be attributable to limitations in clinical pharmacist training in ID and antibiotic stewardship, which has been recognized as an important factor in prior studies.^12,13^ Clinical pharmacist exposure to and training in ID is highly variable, particularly for those without post-graduate training or certification.^13^

Creation, collaboration, and maintenance of institutional guidelines, protocols, and policies related to antibiotics are often the responsibility of an ASP pharmacist which requires familiarity with the most recent ID literature. This may not be the same for clinical pharmacists focused on other areas of practice. Investment in education for front-line clinical pharmacists through tuition assistance programs for ASP/ID-specific continuing education courses can help enrich the ID/ASP educational experience for clinical pharmacists. Likewise, providing institutional resources (eg, institutional guidelines for empiric antibiotic selection) could not only help with an institutional shift to more evidence-based practice, but strengthen antibiotic interventions made by clinical pharmacists.

While we observed a higher incidence of PAFR when PAF followed a clinical pharmacist’s iVent compared to when it did not, the clinical significance of the small absolute difference is uncertain. It is possible that the presence of an iVent is a marker of antibiotic order complexity increasing the likelihood of a subsequent intervention by the ASP. For example, when a provider selects an antibiotic from a condition-specific, EHR-embedded order set, pharmacist-led dose optimization may be needed less often.^14^ Additionally, the order is more likely to be appropriate for the selected condition, reducing the chances of an ASP pharmacist PAFR. The frequency of PAFR types was consistent whether or not there was a prior iVent, supporting the idea that intervention targets and opportunities differ between clinical and ASP pharmacists.

Our single-center experience might not fully mirror practices at other institutions. Given our study’s dependence on documentation of interventions as iVents by clinical pharmacists, it is important to note that documentation may be absent and not reflective of all interventions clinical pharmacists are identifying and communicating. Due to the strict matching procedure requiring that PAF and iVent interventions be performed on the same antibiotic, if an iVent led to a change in antibiotic, the new antibiotic would be categorized as a PAF without a prior iVent. As a result, we may have underestimated the impact of clinical pharmacists’ iVents on preventing future PAFR. Of note, the iVent type to change or discontinue the patient’s antibiotic was uncommon.

Our study highlights the contributions of clinical pharmacists in optimizing antibiotic therapy. The observed differences in intervention types between ASP and clinical pharmacist cohorts may be attributable to the difference in timing and data availability upon antibiotic review. Given their contributions, clinical pharmacists are important ASP ambassadors. Continued investment in clinical pharmacist education and development will be essential to support ongoing antibiotic prescribing improvement efforts.

## Supporting information

Esadah et al. supplementary material 1 Esadah et al. supplementary material

Esadah et al. supplementary material 2 Esadah et al. supplementary material

Esadah et al. supplementary material 3 Esadah et al. supplementary material

## Supplementary material

The supplementary material for this article can be found at https://doi.org/10.1017/ash.2025.69

## Abbreviations

**ASP:** Antimicrobial stewardship program

**FTE:** Full-time equivalents

**iVent:** Clinical pharmacist interventions

**ID:** Infectious diseases

**PAF:** Prospective audit and feedback

**PAFR:** Prospective audit and feedback recommendations

## References

[1] New and Revised Requirements Addressing Antibiotic Stewardship for the Hospital and Critical Access Hospital Programs | The Joint Commission. Accessed August 22, 2022. https://www.jointcommission.org/standards/prepublication-standards/new-and-revised-requirements-addressing-antibiotic-stewardship-for-hospital/

[2] Core Elements of Hospital Antibiotic Stewardship Programs | Antibiotic Use | CDC. Published November 15, 2022. Accessed June 8, 2023. https://www.cdc.gov/antibiotic-use/core-elements/hospital.html

[3] ASHP Statement on the Pharmacist’s Role in Antimicrobial Stewardship and Infection Prevention and Control. Accessed September 9, 2022. https://www.ashp.org/-/media/assets/policy-guidelines/docs/statements/pharmacists-role-antimicrobial-stewardship.ashx 10.2146/sp100001 20237387

[4] DiazGranados CA , Abd TT . Participation of clinical pharmacists without specialized infectious diseases training in antimicrobial stewardship. Am J Health-Syst Pharm AJHP Off J Am Soc Health-Syst Pharm 2011;68:1691–1692. doi: 10.2146/ajhp100482 21880880

[5] Carreno JJ , Kenney RM , Bloome M , et al. Evaluation of pharmacy generalists performing antimicrobial stewardship services. Am J Health-Syst Pharm AJHP Off J Am Soc Health-Syst Pharm 2015;72:1298–1303. doi: 10.2146/ajhp140619 26195656

[6] Barlam TF , Cosgrove SE , Abbo LM , et al. Implementing an antibiotic stewardship program: guidelines by the infectious diseases society of America and the society for healthcare epidemiology of America. Clin Infect Dis 2016;62:e51–e77. doi: 10.1093/cid/ciw118 27080992 PMC5006285

[7] Hurst AL , Child J , Pearce K , Palmer C , Todd JK , Parker SK . Handshake stewardship: a highly effective rounding-based antimicrobial optimization service. Pediatr Infect Dis J 2016;35:1104–1110. doi: 10.1097/INF.0000000000001245 27254036

[8] Suzuki H , Shealy SC , Throneberry K , Stenehjem E , Livorsi D . Opportunities and challenges in improving antimicrobial use during the era of telehealth expansion: a narrative review. Antimicrob Steward Healthc Epidemiol 2021;1:e26. doi: 10.1017/ash.2021.191. PMID: 36168461; PMCID: PMC9495641. 36168461 PMC9495641

[9] ACCP - Board Certification and Recertification. Accessed August 26, 2022. https://www.accp.com/careers/certification.aspx

[10] Bio LL , Kruger JF , Lee BP , Wood MS , Schwenk HT . Predictors of antimicrobial stewardship program recommendation disagreement. Infect Control Hosp Epidemiol 2018;39:806–813. doi: 10.1017/ice.2018.85 29708081

[11] Mas-Morey P , Ballesteros-Fernández A , Sanmartin-Mestre E , Valle M . Impact of clinical pharmacist intervention on antimicrobial use in a small 164-bed hospital. Eur J Hosp Pharm 2018;25:e46. doi: 10.1136/ejhpharm-2017-001307 31157066 PMC6457161

[12] Dionne B , Wagner JL , Chastain DB , Rosenthal M , Mahoney MV , Bland CM. Which pharmacists are performing antimicrobial stewardship: a national survey and a call for collaborative efforts. Antimicrob Steward Healthc Epidemiol 2022;2:e24. doi: 10.1017/ash.2021.245 36310809 PMC9614867

[13] McGee EU , Mason-Callaway AD , Rollins BL . Are we meeting the demand for pharmacist-led antimicrobial stewardship programs during postgraduate training-year 1 (PGY1)? Pharm J Pharm Educ Pract 2020;8:91. doi: 10.3390/pharmacy8020091 PMC7355892 32471109

[14] Nishiguchi JL , Bio LL , Cornell ST , Schwenk HT . Indication-driven order entry decreases stewardship and pharmacist interventions. Infect Control Hosp Epidemiol 2024;45:120–122. doi: 10.1017/ice.2023.155. 37529840

