# Supplementary figures and images for: Untapped potential: exploring clinical pharmacists as antibiotic stewardship ambassadors
Source: Antimicrob Steward Healthc Epidemiol. 2025 Apr 28;5(1):e104. doi: 10.1017/ash.2025.69 (PMC12038757; doi:10.1017/ash.2025.69)

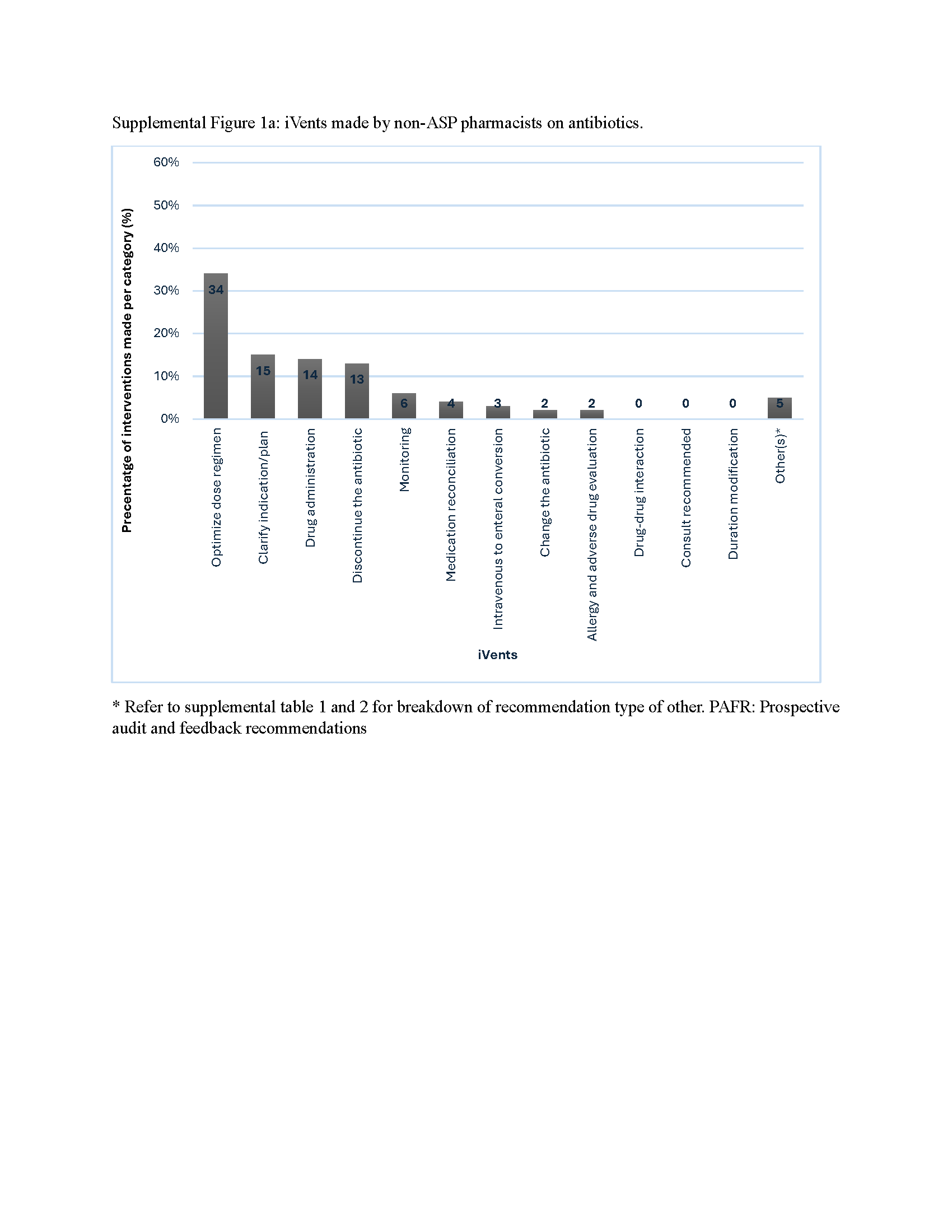

Supplement: Esadah et al. supplementary material 1 — Esadah et al. supplementary material [file S2732494X25000695sup001.tif]

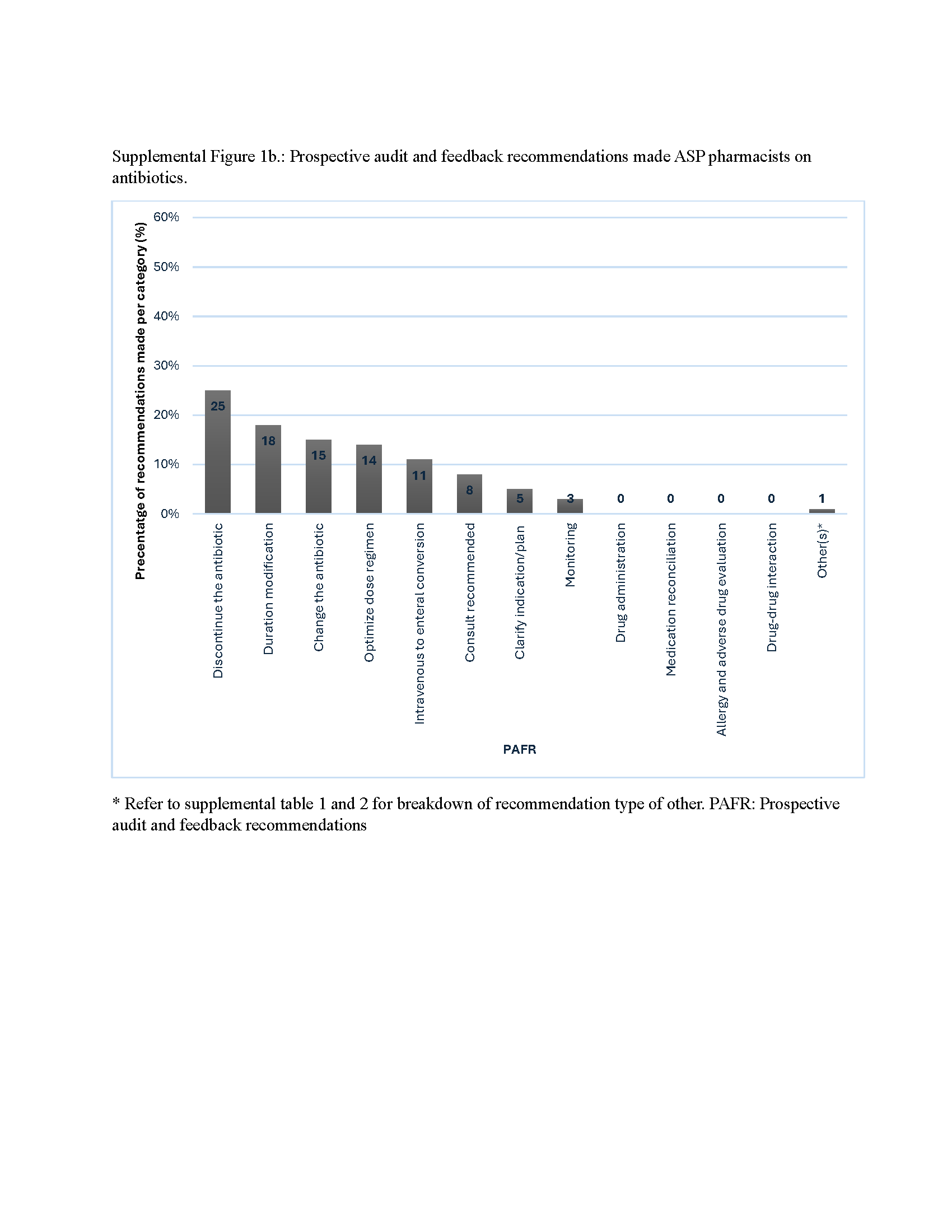

Supplement: Esadah et al. supplementary material 2 — Esadah et al. supplementary material [file S2732494X25000695sup002.tif]
