# Supplementary material for: Untapped potential: exploring clinical pharmacists as antibiotic stewardship ambassadors
Source: Antimicrob Steward Healthc Epidemiol. 2025 Apr 28;5(1):e104. doi: 10.1017/ash.2025.69 (PMC12038757; doi:10.1017/ash.2025.69)
Supplement: Esadah et al. supplementary material 3 — Esadah et al. supplementary material [file S2732494X25000695sup003.docx]

Supplemental Table 1: iVent type and subtype

| Intervention re-coded | Intervention subtypes | Count  n=5,277 |
| --- | --- | --- |
| Allergy and adverse drug evaluation | Allergy classification and documentation | 77 |
|  | Allergy (other) | 31 |
|  | Adverse drug event evaluation | 12 |
|  | Allergy and adverse drug event | 1 |
|  | Adverse drug review | 1 |
|  | | |
| Change antibiotic | Alternative therapy | 99 |
|  | Therapeutic substitution | 17 |
|  | Drug shortage (other) | 7 |
|  | | |
| Clarify order | Order clarification (other) | 410 |
|  | Therapeutic appropriateness (other) | 202 |
|  | Medication indication | 159 |
|  | Non-formulary evaluation | 15 |
|  | Lack of response to standard therapy | 5 |
|  | Order clarification (type = therapeutic appropriateness) | 3 |
|  | Other (type = non-formulary evaluation) | 2 |
|  | Medication evaluation (type = pharmacy consult) | 2 |
|  | Pharmacy consult (other) | 2 |
|  | Non-formulary evaluation | 1 |
|  | | |
| Discontinue | Duplicate therapy | 433 |
|  | Discontinue medication | 193 |
|  | Medication not indicated | 68 |
|  | Contraindication | 2 |
|  | | |
| Drug administration | Date/time clarification | 645 |
|  | Route clarification (type = order clarification) | 53 |
|  | Dosage form evaluation | 17 |
|  | Schedule change | 15 |
|  | Other (type = drug administration recommendation) | 11 |
|  | Compatibility | 8 |
|  | Formulation change (type = value-based utilization) | 7 |
|  | | |
| Drug-drug interaction | Drug-drug | 18 |
|  | Interaction assessment | 3 |
|  | Anticoagulation monitoring | 1 |
|  | Immunosuppressant monitoring | 1 |
|  | | |
| IV to PO | IV to PO | 151 |
|  | | |
| Medication reconciliation | Medication list review | 177 |
|  | Med reconciliation upon admission | 22 |
|  | Medication reconciliation | 5 |
|  | Discharge Medication Evaluation | 2 |
|  | Pharmacy performed medication reconciliation | 1 |
|  | Other (type=medication reconciliation) | 1 |
|  | Patient home medication | 1 |
|  | | |
| Monitoring | Antibiotic monitoring | 229 |
|  | Nephrotoxic monitoring | 35 |
|  | Other (type = therapeutic monitoring) | 29 |
|  | Discontinue lab | 15 |
|  | Decrease lab frequency | 3 |
|  | | |
| Optimize dose regimen | Dose/frequency clarification | 1,180 |
|  | Renal dose adjustment | 245 |
|  | Prolonged infusion | 224 |
|  | Decrease medication frequency | 107 |
|  | Round down dose to nearest vial size | 53 |
|  | Hepatic dose adjustment | 2 |
|  | | |
| Other | Other (type = other) | 126 |
|  | Other (type = family centered rounds pharmacist) | 48 |
|  | Other (type = value-based utilization) | 35 |
|  | Recycle medication (type = value-based utilization) | 26 |
|  | Other (type = intensive care clinical pharmacist) | 15 |
|  | Value-based utilization (type = family centered rounds- pharmacist) | 14 |
|  | Drug information for MDs/NPs/PA (type = drug information and or teaching) | 6 |
|  | Value-based utilization (type = intensive care clinical-pharmacist) | 3 |
|  | Drug information/teaching (Type = family centered rounds-pharmacist) | 1 |

iVents, Clinical Pharmacist Intervention.

Supplemental Table 2: Prospective audit and feedback recommendations type

| ASP recommendation re-coded | Recommendation Type | Count  n= 1782 |
| --- | --- | --- |
| Change antibiotic | Narrow empirically | 121 |
|  | Narrow based on culture/sensitivity | 91 |
|  | Broaden based on culture/sensitivity | 24 |
|  | Broaden empirically | 18 |
|  | Change antibiotic based on culture/sensitivity | 7 |
|  | Change antibiotic (availability) | 4 |
|  | Change antimicrobial (Adverse effect) | 5 |
|  | | |
| Clarify | Clarify indication/plan | 91 |
|  | | |
| IV to PO | IV to PO | 191 |
|  | | |
| Monitoring | Monitoring | 59 |
|  | | |
| Optimize dose regimen | Prolonged infusion (extended or continuous) | 70 |
|  | Increase dose | 55 |
|  | Decrease frequency | 46 |
|  | Increase frequency | 45 |
|  | Decrease dose | 41 |
|  | | |
| Duration modification | Duration suggestion | 154 |
|  | Shorten duration | 154 |
|  | Lengthen duration | 5 |
|  | | |
| Discontinue | Stop (no indication) | 350 |
|  | Stop (redundant therapy) | 80 |
|  | Stop (consolidate to fewer agents) | 13 |
|  | Stop (change to agent with lower frequency) | 2 |
|  | | |
| Consult | ID consult | 145 |
|  | Allergy and immunology consult | 2 |
|  | | |
| Other | Other | 9 |

ASP, Antimicrobial Stewardship Program Pharmacist recommendations.
